# Supplementary material for: Forecasting Trachoma Control and Identifying Transmission-Hotspots
Source: Clin Infect Dis. 2021 Jun 14;72(Suppl 3):S134–9. doi: 10.1093/cid/ciab189 (PMC8201580; doi:10.1093/cid/ciab189)
Supplement: ciab189_suppl_Supplementary-Figures [file ciab189_suppl_supplementary-figures.docx]

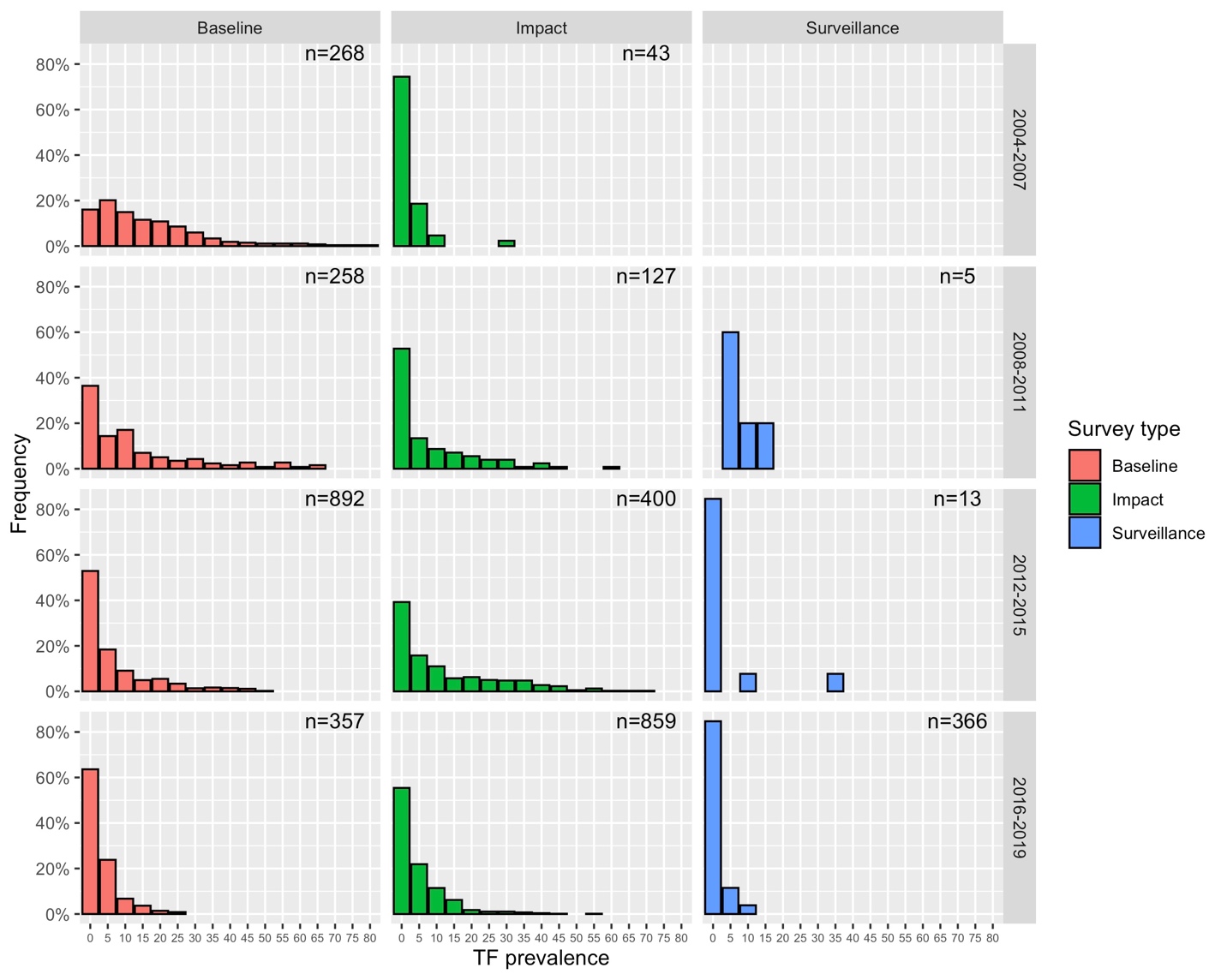


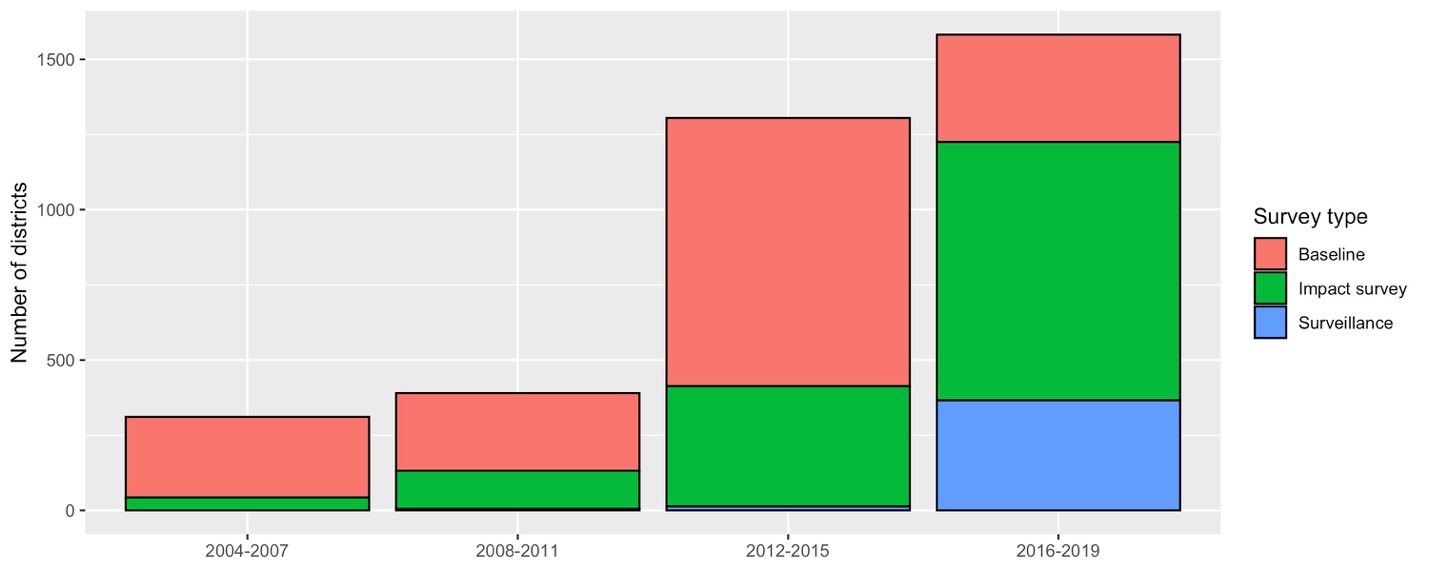


Figure S1: (Top) *Distribution of district-level prevalence of TF as recorded by the GET 2020 alliance* stratified by survey type and four year intervals. (Bottom) Overall number of surveys of each type, grouped by the four year intervals.


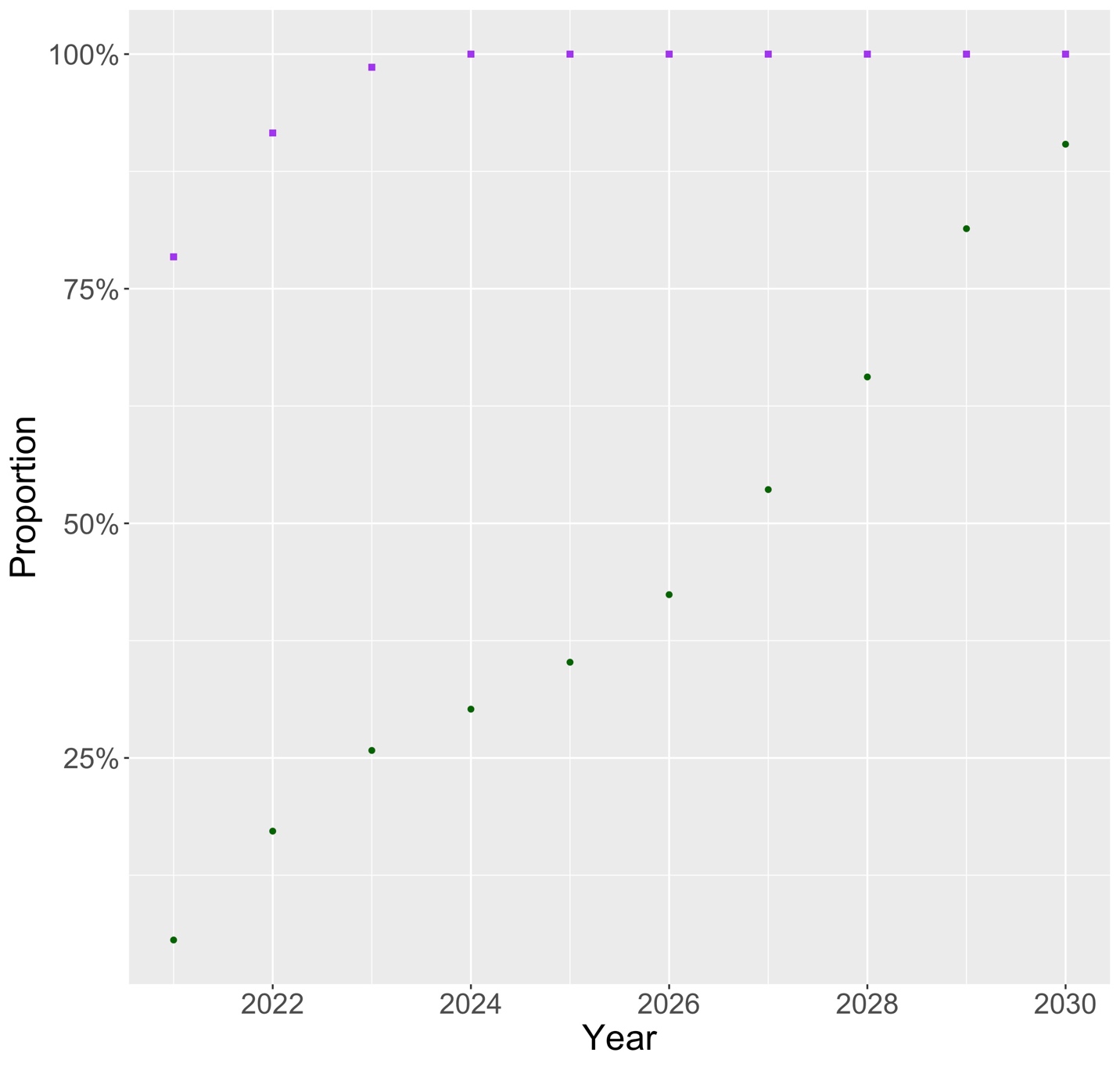


Figure S2: Percentage of bootstrap stimulations that have a mean TF prevalence < 5% (purple squares) and have over 90% of districts with TF < 5% (green circles).


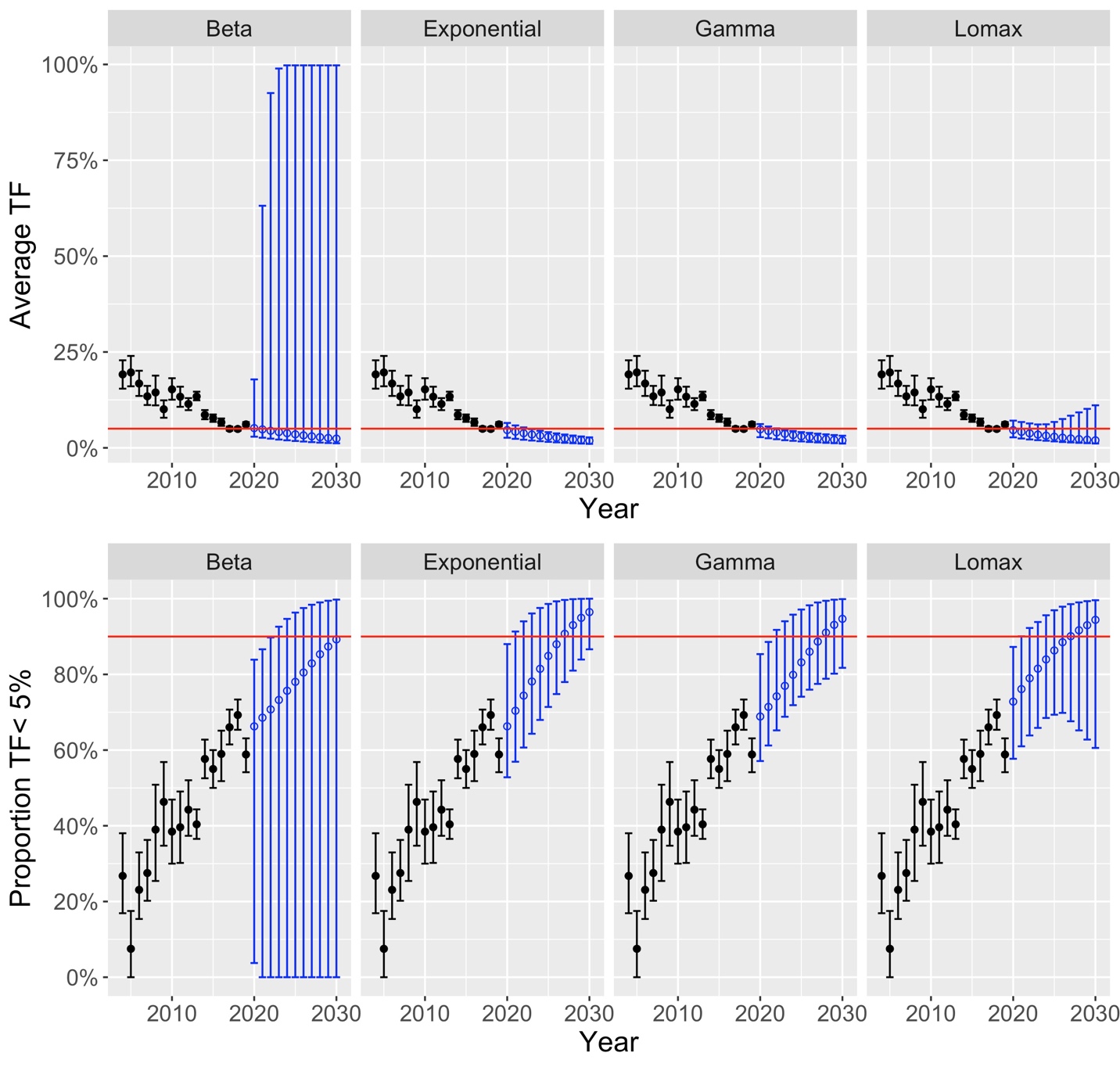


Figure S3: Mean TF prevalence of all surveyed districts (top) and percent of surveyed districts with TF less than 5 percent (bottom). Each vertical column corresponds to a different distribution used for modeling the TF prevalence in any given year. The format of the graphs is identical to that seen if Figure 2 and the panels corresponding to the exponential distribution are a replicate of Figure 2. Additional methodological details concerning the probabilistic forecasts can be found in the Methods Section of the main text.


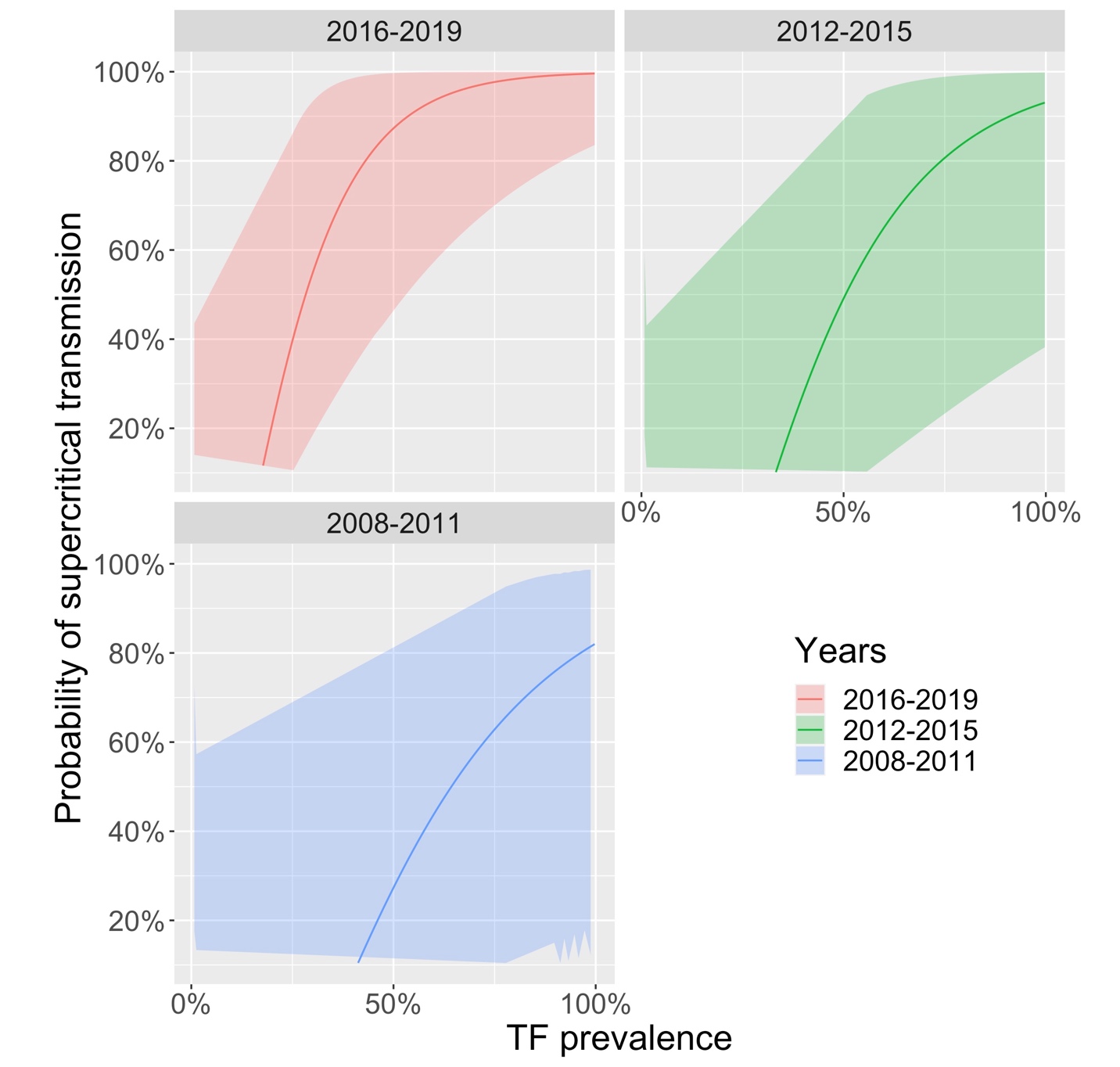


Figure S4: The probability that a district is a transmission-hotspot, as a function of a single TF prevalence measurement. Each panel represents a four-year period. Solid lines are replicates of Figure 3 of the main text and show results for the observed data. The shaded regions are 95% confidence intervals as determined by replicating the analysis on an ensemble of country-level bootstraps.
