# Supplementary material for: Forecasting Trachoma Control and Identifying Transmission-Hotspots
Source: Clin Infect Dis. 2021 Jun 14;72(Suppl 3):S134–9. doi: 10.1093/cid/ciab189 (PMC8201580; doi:10.1093/cid/ciab189)
Supplement: ciab189_suppl_Supplementary-Table [file ciab189_suppl_supplementary-table.docx]

| **Policy Relevant principle** | **Application to manuscript** | **Location of specific detail** |
| --- | --- | --- |
| **Stakeholder engagement** | Study inspired by the GET2020 Alliance. Assessment of trachoma control goals and identification of transmission-hotspots is of key importance to ongoing policy decisions. | Introduction |
| **Complete model documentation** | A complete description of our model is provided. Code is available in a GitHub repository | Methods    Github: @proctor-ucsf/Trachoma-CID-2021-code |
| **Complete description of data used** | Data is obtained from the GET2020 database, as maintained by ITI. | Methods |
| **Communicating uncertainty** | Assumptions of the model that lead to uncertainty in the results are described. Bootstrapping is used to generate confidence intervals | Methods/Discussion |
| **Testable model outcomes** | 1. Forecast of the distribution of TF prevalence for 2020-2030.  2. Probabilistic relationship  between TF prevalence and transmission-hotspots. | Results / Discussion |

***Table S1*** *- Table 2: Summary of Policy-Relevant Items for Reporting Models in Epidemiology of Neglected Tropical Diseases.*[*^35^*](https://www.zotero.org/google-docs/?eeSOH0)

*GET2020 Alliance =  World Health Organization Alliance for the Global Elimination of Trachoma by the year 2020; ITI = International Trachoma Initiative*
